# Manifestation of agronomically valuable traits in the progeny of a sorghum mutant carrying the genetic construct for RNA silencing of the γ-kafirin gene

**DOI:** 10.18699/vjgb-24-08

**Published:** 2024-02

**Authors:** L.A. Elkonin, N.V. Borisenko, T.E. Pylaev, O.A. Kenzhegulov, S.Kh. Sarsenova, N.Yu. Selivanov, V.M. Panin

**Affiliations:** Federal Center of Agriculture Research of the South-East Region, Saratov, Russia; Federal Center of Agriculture Research of the South-East Region, Saratov, Russia; Institute of Biochemistry and Physiology of Plants and Microorganisms, Saratov Federal Scientific Center of the Russian Academy of Sciences, Saratov, Russia Saratov State Medical University named after V.I. Razumovsky, Saratov, Russia; Federal Center of Agriculture Research of the South-East Region, Saratov, Russia; Federal Center of Agriculture Research of the South-East Region, Saratov, Russia; Institute of Biochemistry and Physiology of Plants and Microorganisms, Saratov Federal Scientific Center of the Russian Academy of Sciences, Saratov, Russia; Federal Center of Agriculture Research of the South-East Region, Saratov, Russia

**Keywords:** Sorghum bicolor, transgenic plants, RNA silencing, kafirins, qPCR, grain quality, endosperm texture, Sorghum bicolor, трансгенные растения, РНК-сайленсинг, кафирины, qPCR, качество зерна, текстура эндосперма

## Abstract

Improving the nutritional value of grain sorghum, a drought- and heat-tolerant grain crop, is an important task in the context of global warming. One of the reasons for the low nutritional value of sorghum grain is the resistance of its storage proteins (kafirins) to proteolytic digestion, which is due, among other things, to the structural organization of protein bodies, in which γ-kafirin, the most resistant to proteases, is located on the periphery, encapsulating more easily digested α-kafirins. The introduction of genetic constructs capable of inducing RNA silencing of the γ-kafirin (gKAF1) gene opens up prospects for solving this problem. Using Agrobacterium-mediated genetic transformation of immature embryos of the grain sorghum cv. Avans we have obtained a mutant with improved digestibility of endosperm proteins (up to 92 %) carrying a genetic construct for RNA silencing of the gKAF1 gene. The goal of this work was to study the stability of inheritance of the introduced genetic construct in T2–T4 generations, to identify the number of its copies, as well as to trace the manifestation of agronomically valuable traits in the offspring of the mutant. The mutant lines were grown in experimental plots in three randomized blocks. The studied lines were characterized by improved digestibility of kafirins, a modified type of endosperm, completely or partially devoid of the vitreous layer, an increased percentage of lysine (by 75 %), reduced plant height, peduncle length, 1000-grains weight, and grain yield from the panicle. In T2, a line with monogenic control of GA resistance was selected. qPCR analysis showed that in different T3 and T4 plants, the genetic construct was present in 2–4 copies. In T3, a line with a high digestibility of endosperm proteins (81 %) and a minimal decrease in agronomically valuable traits (by 5–7 %) was selected.

## Abstract (ru)

Улучшение питательной ценности зернового сорго – засухоустойчивой и жаростойкой зерновой культуры, служащей источником кормового и продовольственного зерна во многих засушливых регионах мира, – важная задача селекции в условиях глобального потепления климата. Одной из причин низкой питательной ценности зерна сорго является устойчивость его запасных белков (кафиринов) к протеолитическому расщеплению, обусловленная, в том числе структурной организацией белковых телец, в которых наиболее устойчивый к действию протеаз γ-кафирин располагается по периферии, инкапсулируя более легко перевариваемые α-кафирины. Введение генетических конструкций, способных к индукции РНК-сайленсинга гена γ-кафирина (gKAF1), открывает перспективы для решения этой проблемы. Нами у сорта зернового сорго Аванс посредством агробактериальной трансформации с использованием штамма, несущего в составе Т-ДНК гене- тическую конструкцию для РНК-сайленсинга гена gKAF1, получен мутант с улучшенной перевариваемостью белков эндосперма (до 92 %). Цель настоящей работы – изучение стабильности наследования введенной генетической конструкции в поколениях Т2–Т4, установление числа ее копий, а также проявления важнейших селекционно-ценных признаков у потомства полученного мутанта. Мутантные линии выращивали на экспериментальном участке Федерального аграрного научного центра Юго-Востока в трех рендомизированных блоках. Исследуемые линии характеризовались улучшенной перевариваемостью кафиринов, модифицированным типом эндосперма, полностью или частично лишенным стекловидного слоя, повышенным процентным содержанием лизина (на 75 %), сниженной высотой растений, длиной подметельчатого междоузлия, массой 1000 зерен и урожаем зерна с метелки. В поколении Т2 отобрана линия с моногенным контролем устойчивости к глюфосинату аммония. Анализ qPCR показал, что у разных растений из поколений Т3 и Т4 генетическая конструкция присутствует в двух-четырех копиях. В поколении Т3 отобрана линия с высокой перевариваемостью белков эндосперма (81 %) и минимальным снижением селекционно-ценных признаков (на 5–7 %).

## Introduction

RNA interference (RNAi) is one of the most important natural mechanisms for regulating gene expression and antiviral cell defense. As is known, the mechanism of RNAi is based on the degradation of single-stranded mRNA in the presence of complementary short RNA, which leads to disruption of protein synthesis and silencing of the expression of the corresponding gene. These short interfering RNAs (siRNAs), 20–25 nucleotides long, are transcribed from natural DNA sequences present in the genome or artificially created genetic constructs encoding hairpin RNAs (hpRNAs) (Guo et al., 2016; Muhammad et al., 2019). This design consists of sense and antisense sequences of the target gene mRNA in the form of inverted repeats, which are separated by a spacer sequence. A splicable intron is often used as a spacer in such genetic designs because it improves the efficiency of RNA silencing in plants (Smith et al., 2000).

The sense and antisense sequences in the transcribed RNA are complementary to each other and form hpRNA, which is processed by Dicer-like proteins (DCLs). DCL proteins generate siRNAs from a precursor, hpRNA. One strand of the siRNA duplex is incorporated into the Argonaute protein (AGO), forming the RNA-induced silencing complex (RISC). The siRNA molecule directs RISC to the complementary region of the single-stranded RNA, after which AGO cleaves the target mRNA (Zhuravlyov et al., 2022; Bharathi et al., 2023).

RNAi technology is frequently used in functional genomics and genetic engineering of plants, since it makes it possible to create mutants that are resistant to biotic and abiotic stress factors, as well as to regulate the expression of genes involved in the synthesis and catabolism of important metabolites and reserve nutrients, including proteins, carbohydrates and lipids (Bharathi et al., 2023). This approach has been used to obtain mutants with an altered spectrum of seed storage proteins in cultivated cereal species, including maize, wheat, rice, and sorghum (Elkonin et al., 2016a).

The use of RNAi technology for regulating accumulation of seed storage proteins is of particular importance for sorghum, since it is believed that one of the reasons for the relatively low nutritional value of sorghum grain is the resistance of its storage proteins (kafirins) to proteolytic digestion, due, among other things, to the structural organization of protein bodies, in which the one most stable to the action of proteases, γ-kafirin, is located at the periphery, encapsulating the more easily digestible α-kafirins (Oria et al., 2000; de Mesa-Stonestreet et al., 2010; Duressa et al., 2018). It has been shown that suppression of the synthesis of different classes of kafirins leads to modification of the structure of endosperm protein bodies, a decrease in the resistance of kafirins to protease digestion, and compensatory synthesis of other proteins with a higher lysine content (da Silva et al., 2011a, b; Kumar et al., 2012; Grootboom et al., 2014; Elkonin et al., 2016b).

Previously, through Agrobacterum-mediated genetic transformation, a genetic construct capable of inducing RNA silencing of the γ-kafirin gene (gKAF1), which is a part of the T-DNA vector pNRKAFSIL (Elkonin et al., 2016b), was introduced into the genome of the commercial grain sorghum cultivar Avans. In one of the T0 plants, all kernels had a floury endosperm type and were characterized by a high level of kafirins digestibility (up to 93 %), which was significantly higher than the level of digestibility in the cv. Avans (57 %) (Elkonin et al., 2021). T1 plants inherited these traits, as well as resistance to ammonium glufosinate caused by the bar gene, which was also present in the T-DNA of the pNRKAFSIL vector (Borisenko et al., 2022).

The purpose of this work was to study the stability of inheritance of the introduced genetic construct in T2–T4 generations, its copy number, as well as the manifestation of the most important agronomically valuable traits in the offspring of the mutant, including the amino acid composition of flour and the digestibility of its proteins in an in vitro system.

## Materials and methods

**Fig. 1.**
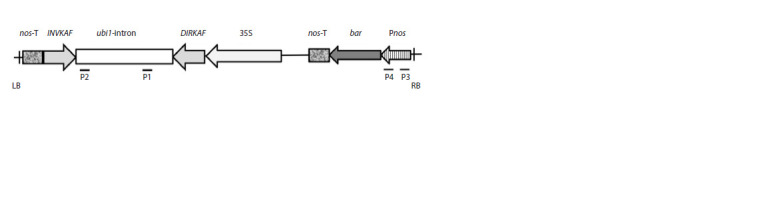
T-DNA of the pNRKAFSIL vector (Elkonin et al., 2016b). DIRKAF, INVKAF – fragments of the sorghum γ-kafirin gene (GenBank Acc. No. M73688) in direct and inverted orientation; maize ubi1-intron; 35S – 35S-promoter of cauliflower mosaic virus; Pnos – nos-promoter; nos-T – nos-terminator; P1–P4 – positions of primers used in PCR analyses.

The original mutant with high digestibility of grain proteins (Avans-1/18) was obtained in an experiment on Agrobacterium- mediated genetic transformation of the new commercial cultivar Avans of grain sorghum (Sorghum bicolor (L.) Moench) using Agrobacterium tumefaciens strain GV3101, carrying the binary vector pNRKAFSIL (Elkonin et al., 2021). This vector contains a genetic construct consisting of a fragment (309 bp) of the γ-kafirin gene (GenBank acc. No. M73688) in direct and inverted orientation, which are separated by the maize ubi1-intron sequence (Fig. 1) (Elkonin et al., 2016b). This construct should suppress gKAF1 expression via RNA interference. The T-DNA of this vector also includes the bar gene under the control of the nos-promoter, which determines resistance to ammonium glufosinate (GA).

The progeny of the RNAi mutant Avans-1/18 was grown in a growth chamber (photoperiod: 16 hours day/8 hours night; t°: 28/22 °С), or in a greenhouse, and in experimental plots in the field of the Federal Center of Agriculture Research of the South-East Region (Saratov, Russia).

To study inheritance of the genetic construct for silencing, panicles of transgenic plants were carefully isolated with parchment bags before flowering. In addition, they were crossed as paternal parents with lines with cytoplasmic male sterility (CMS) A2 KVV-181, A2 KVV-114 and A2 O-1237.

Assessment of resistance to ammonium glufosinate. To study the resistance of the progeny of transgenic plants to GA, the kernels were sterilized with Domestos, then with HgCl2 (0.1 %), after which they were soaked in a GA solution (2.5 mg/l) for 20 hours. After that, the embryos were removed from the soaked kernels and placed on a hormone-free MS nutrient medium containing filter-sterilized GA (2.5 mg/l). The embryos were cultivated in a growth chamber (photoperiod: 16 hours day/8 hours night; t° 26–28 °C) for three weeks. Under this regime, seedlings carrying the bar gene developed normally and reached 10 cm in three weeks, while the development of seedlings not carrying the bar gene was inhibited at the coleoptile growth stage.

Endosperm texture. The texture of the endosperm was determined on transverse sections of mature kernels. The following endosperm types were distinguished: modified, which included the floury type, floury with interspersed vitreous endosperm and floury with a thin, often “blurred” rim of the vitreous layer, and regular endosperm with a thick vitreous layer.

PCR analysis. Genomic DNA was isolated from leaves using a modified CTAB method. The presence of a genetic construct for RNA silencing was checked using PCR analysis with primers that amplified a 588-bp fragment of the maize ubi1-intron (P1 (5′→3′) tgtcttggttgtgatgatgtggtc; P2 (5′→3′): gcgtatgaaggcagggctaaa), which is an important component of RNAi genetic construct, ensuring its stability and functional efficiency (Smith et al., 2000), and the fragment of the nospromoter that controls the bar marker gene, 201 bp long (P3 (5′→3′): tgagactctaattggataccgagg; P4 (5′→3′): tttggaac tgacagaaccgcaac) (primer positions are indicated in Fig. 1). PCR was carried out using DNA amplifiers MasterCycler (Eppendorf, Germany) and T100 (Bio-Rad, USA). PCR conditions were as follows: for the nos-promoter: 95 °C (2 min); 40 cycles [95 °C (30 s), 64 °C (30 s); 72 °C (1 min 10 s)]; 72 °C (7 min). For ubi1-intron: 95 °C (2 min); 40 cycles [95 °C (1 min), 56 °C (1 min), 72 °C (1 min 30 s)]; 72 °C (10 min). The amplified fragments were visualized by electrophoresis in a 2.0 % agarose gel. PCR analyses of each sample were performed in two replications.

qPCR analysis. The copy number of the genetic construct for RNAi was determined using quantitative PCR by amplifying a 119 bp fragment of sorghum anthranilate phosphoribosyl transferase gene (APRT, Sobic.002G303300), selected as a reference gene (Casu et al., 2012; Wang et al., 2021), and a 201 bp fragment of the nos-promoter. The reaction mixture contained 2 μl of genomic DNA (10 ng/μl), 10 μl of a readymade amplification reaction mixture containing the intercalating dye SYBR Green (2X HS-qSYBR-blue, Biolabmix, Russia) and 0.4 pmol of each primer; the total volume of the reaction mixture was 20 μl; the number of replications was three. PCR mode: 1 cycle of 95 °C (2 min), then 40 cycles of [95 °C (15 s), 60 °C (20 s) + detection on the FAM channel, 72 °C (20 s)]. qPCR was performed using a LightCycler 96 real-time PCR instrument (Roche, Switzerland). The primers for the APRT gene were as follows (F (5′→3′)): tgacacattccc aacctcaa and R (5′→3′): atctctctccctgagtggca) (Wang et al., 2021); primers for the nos-promoter are described above. The concentration of genomic DNA and primers was determined using a Nanodrop One C UV spectrophotometer (Thermo Fisher Scientific, USA) by measuring absorbance at wavelengths of 230/260/280 nm. Data analysis and determination of PCR efficiency were performed using the certified LightCycler ® 96 Software, version 1.1.0.1320.

Analysis of total protein content in grain. The total grain protein content of plants from T1 and T2 was analyzed using the Kjeldahl method

Digestibility of grain proteins. To study the digestibility of grain proteins, pepsin treatment of whole-grain flour was used (Aboubacar et al., 2001; Nunes et al., 2004; Wong et al., 2009). A sample of flour (20 mg) was incubated in 5 ml of a 0.15 % pepsin solution (Carl Roth, Germany) (Art.-Nr. KK38.1, 2000 FIP-U/g) in 0.1 M potassium phosphate buffer (pH 2.0) for 120 min at 37 °C with occasional shaking.

To quantify digestibility, a method based on scanning the electrophoretic spectra of proteins obtained in SDS-PAGE was used (Aboubacar et al., 2001; Wong et al., 2009; Elkonin et al., 2013). Proteins were isolated from digested and control samples using extraction buffer (0.0625 M TRIS·HCl, 2 % SDS, 5 % 2-mercaptoethanol, pH 6.8). Samples were subjected to SDS-PAGE in 12.5 % (w/v) PAGE; 10 μl of extract was applied to each lane. Protein molecular weight markers (14–116 kDa) (Thermo Scientific) were used as standards. Gels were stained with Coomassie Brilliant Blue R-250. After electrophoresis, the gels were scanned using ChemiDocTM (Bio-Rad); protein amounts were determined using Image Lab 6.1 (Bio-Rad). The digestibility value was calculated as the percentage of the difference between the volume of protein in the control sample and in the sample treated with pepsin to the volume of protein in the control sample. The experiments were performed in two replications

HPLC analysis of the amino acid composition of flour proteins. To analyze the total content of amino acids, 10 kernels were examined from transgenic plants T1 190-1, 190- 3, 190-4 and the original cv. Avans. Ground meal samples (15 mg) were hydrolyzed with 1.5 ml of 6N HCl (at t° of 106 °C for 24 h) under nitrogen. Identification of amino acids was carried out using a pre-column modification with 6-aminoquinolyl-N-hydroxysuccinimidyl carbamate according to the Waters AccQ-Tag method using a WAT 052880 reagent kit. The analysis was carried out by HPLC on a Knauer Smartline chromatograph using reverse phase chromatography on a Kromasil – 110 C18/2.5 μkm (2 mm × 150 mm). Detection was performed at λ 248 nm. The injection volume was 20 μl. Samples were analyzed in triplicate. Quantitative calculation of the amino acid content in sample hydrolysates was carried out using the external standard method – 250 pM of analytical amino acid standard (AAS18 Fluka).

Evaluation of agronomically valuable traits. To study the influence of the RNAi genetic construct for silencing the gKAF1 gene on the manifestation of agronomically valuable traits, the T3 and T4 progeny of four T1 transgenic plants, as well as the original Avans variety, were grown in 4 meter rows in three replications in the experimental field of the Federal Centre of Agriculture Research of the South-East Region. All panicles of plants were carefully bagged with parchment bags before flowering. The following traits were analyzed: plant height, peduncle length, weight of 1000 grains, and grain yield per panicle. In each replication, the average value of the trait was determined in 10–15 plants.

Methods of biological statistics. To assess differences in the in vitro digestibility of proteins of the studied samples, dispersion analysis and Duncan’s Multiple Range Test were performed using the AGROS software package, version 2.09 (S.P. Martynov, Institute of General Genetics of RAS). Data on the manifestation of agronomically valuable traits were processed by one-way dispersion analysis using the AGROS software package

## Results and discussion

PCR analysis of plants from T2–T4 generations with primers to the ubi1-intron separating inverted fragments of the gKAF1 gene in the pNRKAFSIL vector, and to the nos-promoter that controls the expression of the bar gene, showed the stability of inheritance of the genetic construct during self-pollination. In the T2 generation, the progeny of four T1 plants was analyzed, each of which was PCR-positive for both loci studied (Nos. 1-3, 190-1, 190-2, 190-4), as well as the progeny of plant No. 190-3, which was PCR negative for the ubi1-intron, but PCR positive for the nos-promoter (Elkonin et al., 2021).

It was found that all 12 T2 plants from the T1 family No. 1-3 and all 8 T2 plants from the T1 family No. 190-4 were PCR positive for both loci tested (Table 1). At the same time, in the T2 families – the progeny of plants 190-1 and 190-2 – segregation was observed, and PCR-negative plants for both loci were present. No PCR-negative plants were found in the T3 and T4 generations. This fact indicates the stability of inheritance of the introduced genetic construct

**Table 1.**
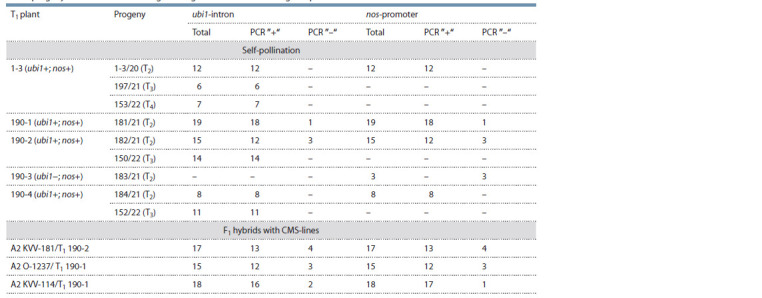
Inheritance of the genetic construct for RNA silencing of the gKAF1 gene in the progeny of the RNAi mutant of grain sorghum Avans-1/18 during self-pollination and in crosses with CMS lines

PCR analysis of F1 hybrids between T1 plants and different CMS lines showed the possibility of transmitting the introduced genetic construct through pollen. It is noteworthy that among the F1 hybrids with the CMS line A2 KVV-114, one plant that was PCR positive for the nos-promoter but PCR negative for the ubi1-intron was found. We previously found a similar case among T1 plants (Elkonin et al., 2021). These facts indicate that in some cases there may be a disturbance of the integrity of the integrated T-DNA and the elimination of the genetic construct for silencing. A significant predominance of PCR-positive plants in T2, as well as among F1 hybrids, indicated the presence of several unlinked copies of the genetic construct in T1 plants

Resistance to ammonium glufosinate. To further study the inheritance of the genetic construct for RNA silencing, the progeny of a number of plants from T1–T3 generations was grown on a nutrient medium containing ammonium glufosinate (2.5 mg/l), resistance to which is determined by the presence of a bar gene. In different plants, different segregation patterns were observed: 15:1 (plant No. 182-3), indicating the presence of two copies of the genetic construct; monogenic segregation 3:1 (No. 124-3; 124-3-9; 124-3-10), indicating the presence of one copy of the construct; in the progeny of plants No. 150-15, 124-3-3, 190-4, no segregation was observed, which suggested homozygous nature of these plants (Table 2). Considering that the plant 124-3-3 was obtained from the progeny of plant 124-3, which segregated as a monoheterozygote (3:1), it is quite obvious that in the plant 124-3-3, the genetic construct for the silencing of the gKAF1 gene is present in one copy in a homozygous state. No resistant plants were observed in the progeny of plant 190-3, as well as in the original cv. Avans.

**Table 2.**
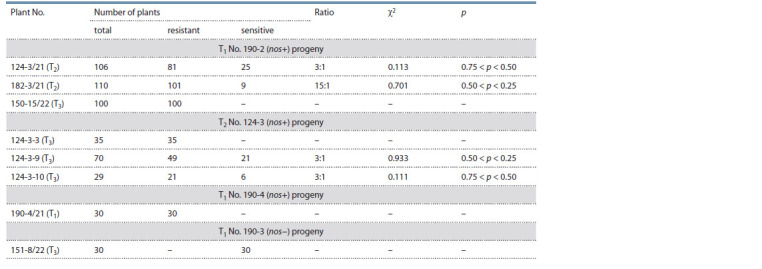
Segregation for resistance to ammonium glufosinate in the progeny of some plants of the Avans-1/18 mutant carrying genetic construct for RNA silencing of the gKAF1 gene

**Fig. 2.**
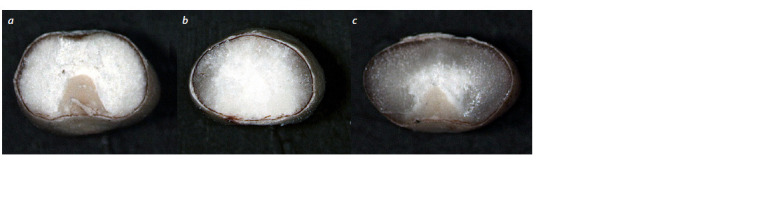
Cross sections of kernels from the progeny of the RNAi grain sorghum mutant Avans-1/18 (a, b) and the original non-transgenic cv. Avans (c). A thick layer of vitreous endosperm is noticeable in the kernels of the original cultivar (c) and there is a complete absence of vitreous endosperm (a) or a blurred layer of vitreous endosperm in the kernels of transgenic T1 plants (b).

Endosperm texture. Analysis of the endosperm texture in kernels of plants from different generations of the RNAi mutant Avans-1/18 showed the inheritance of a modified endosperm type (floury or floury with vitreous inclusions) (Fig. 2), characteristic of the mutant kernels, up to the T4 generation. In the panicles of T1 and T2 plants, due to their heterozygosity, a 3:1 or 15:1 segregation (modified endosperm type: normal vitreous endosperm) was observed, which indicated the presence of one or two copies of the genetic construct (Table 3). In the T3 and T4 generations, segregation of the kernels with vitreous endosperm was absent, as a rule, indicating the homozygous state of the introduced genetic construct.

**Table 3.**
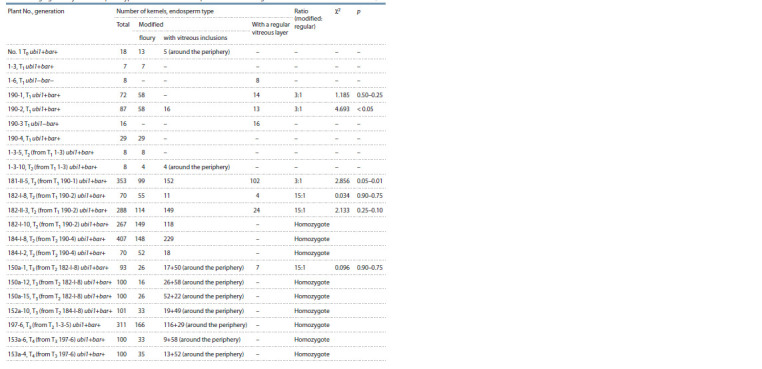
Segregation by the endosperm type in the kernels of some plants from different generations of the RNAi-mutant Avans-1/18

It is noteworthy that in many kernels a combined type of endosperm was observed, in which the floury endosperm was interspersed with sectors of vitreous endosperm, or the vitreous endosperm was present in the form of a thin, often “blurred” peripheral layer (see Fig. 2). The formation of these types of endosperms did not depend on the number of copies of the construct (see below) and, possibly, reflected the peculiar properties of its expression under environmental conditions that change during kernel maturation.

Similar “combined” endosperm types were described previously in other works on the introduction of genetic constructs for silencing kafirin genes (da Silva, 2012; Elkonin et al., 2016b), as well as in recombinant lines obtained by hybridization of the P721Q mutant, characterized by high digestibilityof kafirins and floury endosperm, with common sorghum lines with low kafirin digestibility and regular vitreous endosperm (Tesso et al., 2006). Considering that the floury endosperm type is accompanied by a number of negative agronomic traits (Duressa et al., 2018), lines with such combined endosperm types and improved protein digestibility should have higher breeding value. 

Analysis of the number of copies of RNAi genetic construct. To clarify the copy number of the genetic construct for silencing of gKAF1 in T3 and T4 plants, quantitative PCR analysis (qPCR) was carried out with primers to the nos-promoter, while the sorghum APRT gene, which was previously used in experiments to identify the number of copies of transgenes in different plant species, including sorghum (Casu et al., 2012; Wang et al., 2021), was used as a reference control. In addition, the copy number of the introduced genetic construct was also calculated using DNA from the plant No. 124-3-9 (T4), which was segregated as a mono-heterozygote (3:1), was also used as a reference control (see Table 2).

It was found that in those plants that, based on the results of segregation analysis for the bar gene, were expected to have one copy of the genetic construct, the copy number, according to qPCR data, was the lowest (0.6...0.9; on average 0.76 ± 0.06) (Table 4). At the same time, in the plant No. 124- 3-3 – a putative homozygote with two copies of the genetic construct – the copy number, according to qPCR data, was 2 times higher (1.4 ± 0.2). These data show that our qPCR analysis quite accurately reflects the copy number of the construct under study, and the results obtained using it are trustworthy.

**Table 4.**
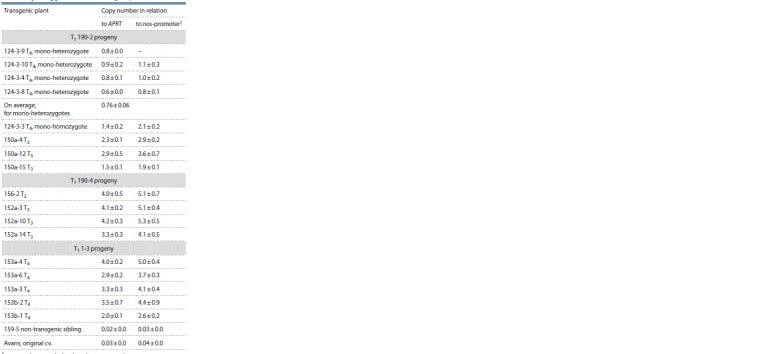
The number of copies of the genetic construct for RNA silencing of the gKAF1 gene in different plants from the T3 and T4 generations according to qPCR results Copy number was calculated in relation to mono-heterozygote, No. 124-3-9 (Т4).

The analysis revealed variations in the number of copies of the genetic construct for RNA silencing in different T3 plants. Thus, plant No. 150a-15 from the T1 190-2 family had two copies of the construct (see Table 4). Considering the lack of segregation for the bar gene (see Table 2) and for the endosperm type in the kernels formed on its panicle (see Table 3), it is obvious that it is a mono-homozygote. At the same time, in the plant No. 150a-12, the copy number was significantly higher, indicating the presence of three-four copies of the genetic construct. The presence of individuals with two and four copies of the construct from the progeny of the same T1 plant (190-2) indicates the presence of two independent T-DNA insertions. This assumption is confirmed by the di-hybrid segregation pattern (15:1) for the bar gene and for the endosperm type in the progeny of some plants from the 190-2 family.

Thus, the data of classical genetics and qPCR quite accurately agree with each other and indicate the presence of at least two independent copies of the genetic construct for RNA silencing in the parental T0 transgenic plant. Noteworthy is the high copy number of the construct in plants from the progenies of T1 190-4 and 1-3 plants in T3 and T4 generations, which carried at least four copies of the genetic construct.

**Fig. 3.**
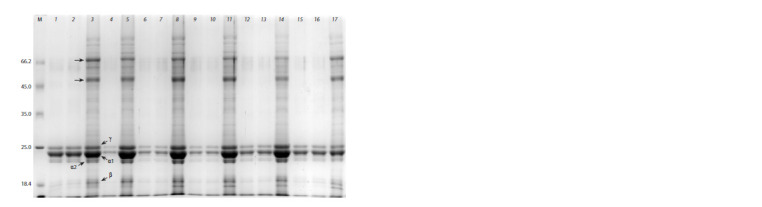
SDS-PAGE of grain proteins of the original cv. Avans and plants of Avans-1/18 after pepsin treatment of the flour. 1–3, 15–17 – original cv. Avans; 4, 5 – Т1 190-4; 6–11 – Т2 184-I-8 (progeny from 190-4); 6–8 – grains with floury endosperm; 9–11 – grains with floury endosperm interspersed with vitreous endosperm; 12–14 – Т2 184-I-7 (progeny from 190-4) – grains with pink floury endosperm. 1, 2, 4, 6, 7, 9, 10, 12, 13, 15, 16 – after treating of the flour with pepsin; 3, 5, 8, 11, 14, 17 – without pepsin treatment (control). The positions of different classes of kafirins are marked with arrows. Dotted arrows indicate proteins belonging to the globulin fraction.

In vitro digestibility of grain proteins. An assessment of the digestibility of grain proteins obtained from wholeground kernels set on panicles of T1 and T2 plants showed the inheritance of a high level of protein digestibility found in T0 generation, although the values in most T2 plants were slightly lower than the values of this trait in T1. Plants grown with artificial watering during grain maturation (2 times per 7–8 l/m2) had higher digestibility compared to plants grown in the absence of additional watering (Table 5). Apparently, conditions of higher water availability contribute to more efficient expression of the genetic construct for silencing. Kernels with a floury endosperm type and with endosperm containing inclusions of vitreous endosperm had the same level of protein digestibility (Fig. 3). At the same time, kernels with a regular vitreous endosperm had lower digestibility compared to kernels with a mutant type of endosperm (floury or with vitreous inclusions).

**Table 5.**
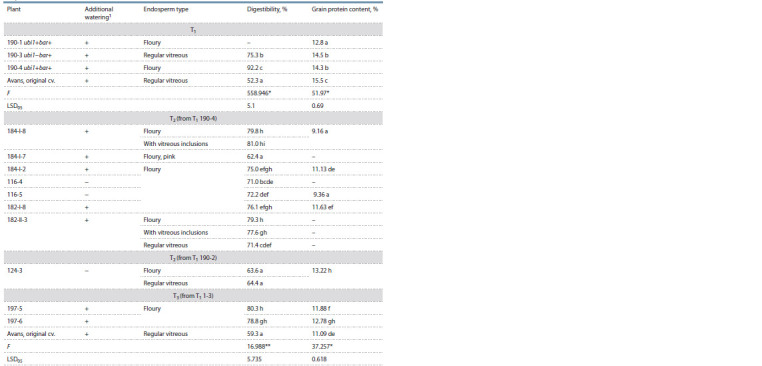
In vitro grain protein digestibility in plants from the progeny of the Avans-1/18 mutant carrying the genetic construct for RNA silencing of the gKAF1 gene Additional watering: 2 times per 8 l/m2 during the period of grain maturation; * p < 0.05, ** p < 0.01; data denoted by different letters significantly differ at p < 0.05 according to Duncan’s Multiple Range Test.

Remarkably, in the plant 184-I-7, an unusual floury type endosperm with a pink coloration was found. This coloration is possibly conditioned by polyphenols, which normally determine the color of the vitreous layer of the endosperm in the kernels of the original cv. Avans. The flour obtained from the same plant had a similar coloration. The digestibility of proteins in such flour was slightly lower than that of flour from other plants, possibly due to the inhibition of pepsin action by polyphenols.

The total protein content in the grain of T1 plants decreased by 7.5–17.3 % compared to the original cv. Avans: 12.8–14.3 % vs. 15.5 %, while the percentage of lysine in the grains of plant No. 190-4 increased by 75 % (see Supplementary Material)1, which may have been a consequence of re-balancing protein synthesis in the grains of transgenic plants and the appearance of proteins with higher lysine content compared to γ-kafirin. This re-balancing of protein content has been previously described in many cereal RNAi mutants with genetic constructs that suppress prolamine synthesis (Elkonin et al., 2016a). The protein content in the grain of different T2 plants varied from 9.2 to 12.8 % and in most of the studied plants it did not differ significantly from the original cv. Avans (11.1 %). In two plants from family No. 190-4 (184-I-8 and 116-5), apparently containing two copies of the genetic construct, the protein content was significantly lower (9.2–9.4 %). However, the influence of the number of copies of the genetic construct for silencing the gKAF1 gene on the total protein content in grain requires additional investigation.


Supplementary Materials are available in the online version of the paper: https://vavilov.elpub.ru/jour/manager/files/Suppl_Elkonin_Engl_28_1.pdf


Analysis of agronomically valuable traits. To study the manifestation of agronomically important traits in the plants carrying the genetic construct for RNA silencing of the γ-kafirin gene, in 2020, a few T1 plants were grown in an experimental plot under field conditions. It was found that these plants have a reduced height compared to the original cv. Avans: 82.3 ± 1.7 cm and 100.8 ± 3.4 cm, respectively ( p < 0.01). These differences were also observed in T2 generation: 81.5–91.6 cm, on average, in different T2 families, compared to 104.1 cm for the original cultivar ( p < 0.05) (2021 data). In this connection, for a more detailed study of the manifestation of the main agronomically valuable traits in the mutant, T3 families (the progeny of three T1 plants Nos. 190- 2, 190-3, 190-4, which were grown in the field), and one T4 family (the progeny of T1 plant No. 1-3 from the greenhouse) were grown in 2022 in experimental plots, in 3 replications.

It was found that all the studied lines of transgenic origin derived from a single initial T0 plant differ from the original cv. Avans in reduced plant height, shortened peduncle and reduced grain yield per panicle (Table 6). Such a decrease in the manifestation of these traits in mutants carrying genetic constructs for RNA silencing of prolamin genes is described for the first time in cereals. Such a change in plant height and peduncle length may be a consequence of off-target effects of expression of a genetic construct for RNA silencing, which are well known and described in the literature (Jackson et al., 2003; Senthil-Kumar, Mysore, 2011). However, this explanation seems unlikely since in the line No. 151 (progeny of plant 190-3), this construct was eliminated, while the short stature and shortened peduncle were preserved.

**Table 6.**
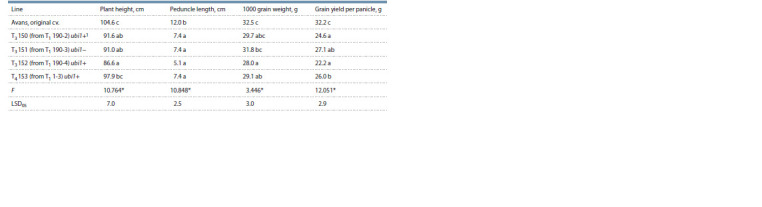
Manifestation of some agronomically important traits in the progeny of the mutant Avans-1/18 (averages from three replications) The presence of the genetic construct for RNA silencing of the gKAF1 gene (based on the results of PCR with primers for the ubi1-intron). Data denoted by different letters are significantly different at p < 0.05, according to Duncan’s Multiple Range Test.

Another explanation may be the insertion of the genetic construct for RNA silencing into one of the loci that controls plant height and peduncle length. Similar facts of induction of mutations in genes encoding various plant traits as a result of T-DNA insertion are widely known (Wilson et al., 2006; Deineko et al., 2007; Ram et al., 2019). In most cases, such insertions lead to plant DNA deletions that disrupt the functional activity of genes. Previously, mutations of short stature caused by T-DNA insertion, including mutation of the locus that controls gibberellin synthesis, which is involved in the regulation of plant height, were described in Arabidopsis (Feldmann et al., 1989; Chiang et al., 1995). In sorghum, we were unable to identify any reports on the induction of mutants by T-DNA insertion in the available literature.

The weight of 1000 grains was significantly reduced in two lines carrying the RNA-silencing construct (Nos. 152, 153). Obviously, such a decrease in the weight of 1000 grains is a consequence of modification of the endosperm type, since in the loose floury endosperm, there are air cavities between the starch granules. It is noteworthy that in line No. 151 (progeny of plant 190-3), in which the kernels, due to the elimination of the RNA-silencing construct, had a regular endosperm with a thick vitreous layer, there were no significant differences for this trait from the original cv. Avans. The absence of significant differences between the original cultivar and line No. 150 is also possibly explained by the presence of heterozygous plants, the panicles of which contain kernels with vitreous endosperm (see Table 3). Thus, the decrease in 1000-grain weight is a direct consequence of the expression of the gKAF1 gene RNA-silencing construct. A similar decrease in 1000-grain weight was described in sorghum RNAi mutants obtained in the African variety P898012, carrying a genetic construct for RNA silencing of two γ-kafirin genes, 27 and 50 kDa (Ndimba et al., 2017).

Such a decrease in grain weight could not but cause a decrease in grain yield per panicle, which was observed in all transgenic lines we obtained, and in this regard, a change in this trait is also a consequence of the expression of the introduced genetic construct. At the same time, the drop in grain yield may also be due to a reduced development power of plants due to T-DNA insertion into one of the loci that controls plant height, since this trait was also reduced in line No. 151, in which the silencing construct was eliminated. In this regard, a decrease in grain yield per panicle may also be a consequence of insertion mutagenesis induced by T-DNA.

It should be noted that the presence of several unlinked copies of the genetic construct, apparently integrated into different regions of the genome, which, due to the effect of gene position, may differ in their effect on the phenotypic traits of plants, opens up the possibility of selecting lines that combine improved protein digestibility with the required manifestation of agronomically valuable traits. Thus, a line was identified (No. 153, T4 derived from T1 1-3), in which plant height and grain yield per panicle were reduced to a lesser extent (by 5–6 %), while the level of protein digestibility reached 81 % (see Table 5). In this regard, this line may be of greater interest for practical breeding.

## Conclusion

The results of this study indicate that the genetic construct for RNA silencing of the sorghum γ-kafirin gene (gKAF1) is represented in the genome of different plants from T3 and T4 generations of the RNAi mutant Avans-1/18 in 2–4 copies, which, apparently, are a consequence of two independent events of T-DNA integration. This construct is stably inherited and expressed in the T3 and T4 generations, modifying the endosperm texture, improving protein digestibility (up to 81 %, compared to 52–59 % in the original cultivar), and increasing the percentage of lysine (by 75 %). At the same time, cases of disturbances of the integrity of the integrated T-DNA and elimination of the genetic construct for silencing have been recorded. Transgenic plants of the RNAi mutant Avans-1/18 are characterized by reduced plant height, shortened peduncle, reduced 1000-grain weight, and panicle grain yield. A line (T3) with high digestibility of endosperm proteins (81 %) and a minimal decrease in agronomically valuable traits (by 5–7 %) was isolated.

## Conflict of interest

The authors declare no conflict of interest.
